# Extracellular Cr(VI) Reduction by the Salt-Tolerant Strain *Bacillus safensis BSF-4*

**DOI:** 10.3390/microorganisms13081961

**Published:** 2025-08-21

**Authors:** Yilan Liu, Weiping Yu, Tianying Nie, Lu Wang, Yusheng Niu

**Affiliations:** 1 Institute of Biomedical Engineering, College of Life Sciences, Qingdao University, Qingdao 266071, China; liuyilan2022@163.com (Y.L.); ying18854709906@163.com (T.N.); 2 Research Institute of Modern Agricultural Industry Innovation in Yellow River Delta Saline-Alkali Land, Dongying Vocational College, Dongying 257029, China; adongzi@126.com

**Keywords:** *Bacillus safensis*, hexavalent chromium, reduction mechanism, transcriptomics

## Abstract

Microbial reduction in hexavalent chromium (Cr(VI)) is a well characterized bioremediation strategy, yet the mechanistic diversity among bacterial taxa necessitates detailed investigations into strain-specific pathways. Here, we report the isolation and characterization of *Bacillus safensis BSF-4*, a halophilic bacterium derived from saline-alkali soil, which demonstrates efficient Cr(VI) reduction capacity. Physiological assays showed that *BSF-4* achieved 89.15% reduction of 20 mg/L Cr(VI) within 72 h, with Cr(III) identified as the primary extracellular end product. Resting cell assays and subcellular fractionation analyses confirmed that Cr(VI) reduction predominantly occurs in the extracellular milieu. X-ray photoelectron spectroscopy (XPS) further revealed soluble Cr(III) complexed with extracellular polymeric substances (EPS). Transcriptomic profiling indicated upregulation of membrane-associated transport systems (facilitating Cr(VI) exclusion) and quorum sensing (QS) pathways (mediating adaptive stress responses). These findings highlight a dual mechanism: (1) extracellular enzymatic reduction mediated by EPS-bound redox proteins, and (2) intracellular detoxification via QS-regulated defense pathways. Collectively, *Bacillus safensis BSF-4* exhibits robust Cr(VI) reduction capacity under saline conditions, positioning it as a promising candidate for bioremediation of Cr(VI)-contaminated saline soils and aquatic ecosystems.

## 1. Introduction

Heavy metal pollution is constantly affecting the environment and threatening human health [1]. The widespread Cr(VI) pollution in soil and water has become a serious public health problem [2,3]. Chromium occurs naturally in environmental systems with oxidation states spanning from −II to +VI, with the predominant aqueous species existing as hexavalent chromate Cr(VI) and trivalent chromium Cr(III) ions [4]. Cr(VI) has higher mobility, solubility, and toxicity than Cr(III) [5], which has been classified as one of the priority pollutants due to its carcinogenicity, teratogenicity, and mutagenicity to living organisms [6,7]. In China, the accumulation of approximately 67 million tons of chromium waste has resulted in 7 percent of the country’s soil containing chromium levels that exceed environmental quality standards [8]. The general pollutant limit for Cr in China’s “Soil Environmental Quality Standard (GB15618-2018)” [9] is set at 350 mg/kg. The permissible concentration of Cr(VI) in water bodies is generally much lower than in soil. According to China’s “Surface Water Environmental Quality Standard” [10], the maximum allowable concentration of Cr(VI) in surface water is 0.05 mg/L. The U.S. Environmental Protection Agency (EPA) has promulgated an enforceable maximum contaminant level (MCL) of 0.1 mg/L for Cr(VI) in public water systems under the National Primary Drinking Water Regulations (NPDWR) [11]. Cr(VI) seeps into groundwater from contaminated soil, causing serious contamination to water systems [12]. Many chromium-containing industrial wastewater have a high salt content, and most Cr(VI)-reducing bacteria have limited reducing capacity under a high salt environment [13,14]. Therefore, the discovery of more Cr(VI)-removal microorganisms that can work in high salt environments, such as saline soil, is of great significance for reducing Cr(VI) pollution in the environment.

At present, physical and chemical methods for removing Cr(VI) from the environment include adsorption [15], flocculation and precipitation [16] and electrochemical methods [17], as well as bioremediation. Among them, physical and chemical methods exhibit technical merits such as high treatment efficiency and facile operation; they are generally plagued by limitations, including substantial equipment investment, high operational costs, and risks of secondary pollution, while the bioremediation strategy has the advantages of low cost and environmental friendliness [18,19]. Bioremediation has emerged as a novel strategy for enzymatic reductive detoxification of hexavalent chromium, serving as a sustainable alternative to conventional physicochemical treatment technologies by offering enhanced eco-efficiency and reduced secondary contamination risks. Microbial consortia demonstrate particular promise in chromium bioremediation owing to their phylogenetic diversity, metabolic versatility, and niche adaptability, enabling efficient Cr(VI) reduction through both direct enzymatic and indirect electron-shuttling mechanisms [20,21].

So far, scientists have identified a number of strains that can specifically reduce Cr(VI). For example, in the presence of 5 mg/L Cr(VI), suspension cells of *Arthrobacter* sp. Sphe3 can achieve a Cr(VI) reduction rate of 94% [22]. *Bacillus cereus* was able to reduce all Cr(VI) with an initial mass concentration of 10~50 mg/L, which laid a foundation for the research on chromium reduction [23]. *Aspergillus niger* can remove 70% of 500 mg/kg of chromium from soil [24]. *Serratia marcescens* was isolated and screened from chromium-contaminated land, and the Cr(VI) tolerance mass concentrations were up to 1000 mg/L [25,26]. It was found that the mechanisms of specific reduction in Cr(VI) by these strains were diverse and diversified, which were mainly divided into two categories: enzyme-catalyzed reduction and non-enzymatic catalytic reduction [27]. Non-enzyme-catalyzed reduction is a process of reduction mediated by reduced compounds produced by microbial growth and metabolism, such as glutathione and amino acids [28]. This non-specific pathway is particularly prevalent among anaerobic microorganisms, which often utilize Cr(VI) as a terminal electron acceptor in anaerobic respiration processes. For example, sulfate-reducing bacteria like *Desulfovibrio vulgaris* can couple Cr(VI) reduction to organic matter oxidation in anoxic environments, leveraging metabolic byproducts like hydrogen sulfide for indirect chromate reduction [29]. Most microorganisms use enzyme-catalyzed reduction methods, such as ferric ion reductase, nitro reductase, flavin reductase, dehydrogenase, and NAD(P)H-dependent reductase, etc. [30]. However, these reduction mechanisms in previous research are not comprehensive enough. There are still many aspects that remain unclear, such as the detailed reaction pathways. Therefore, the present investigation employs an integrated molecular–cellular approach combined with transcriptomic profiling to delineate the underlying regulatory mechanisms at multiple biological levels.

In this study, a strain of halotolerant bacterium *Bacillus safensis BSF-4* with Cr(VI) reduction has been screened and isolated from saline soil. By exploring the tolerance and reduction capacity of this strain to Cr(VI), it was demonstrated that at an initial Cr(VI) concentration of 20 mg/L, the reduction efficiency of Cr(VI) is as high as 89.15% within 72 h. In the process of reducing Cr(VI), Cr(VI) acts as an electron acceptor and NAD(P)H or cytochrome proteins as electron donors [27] to achieve electron transfer from Cr(VI) to Cr(III) under the catalysis of intracellular, cell membrane, or extracellular reductase. At the same time, the strain increased the expression of the degrading enzyme by quorum sensing to resist the stress of the hexavalent chromium toxicant on cells, which belonged to enzyme-catalyzed reduction. Given the prevalence of high-salt chromium-contaminated environments (e.g., saline soils and industrial wastewater) and the limited efficiency of most reported Cr(VI)-reducing microorganisms under such conditions, the isolation of *Bacillus safensis BSF-4* fills a critical gap in microbial remediation resources for extreme environments. By expanding the Cr(VI)-reducing bacterial library and investigating the reduction mechanism of *Bacillus safensis BSF-4*—including identifying the primary reduction sites—this study provides a theoretical basis and potential microbial resources for the bioremediation of Cr(VI)-contaminated soil and groundwater, particularly in high-salt settings.

## 2. Materials and Methods

### 2.1. Isolation, Identification, and Preservation of the BSF-4 Strain

Saline soil samples were collected from Binzhou, Shandong Province, China (37.22° N, 118.02° E). Take 5 g of soil sample into a 250 mL sterilized conical flask, add 75 mL of distilled water and place it on a constant temperature shaker at 35 °C, 200 rpm for one hour, then take 5 mL of soil suspension and centrifuge it into a centrifuge tube (6000 rpm, 5 min), take 2 mL of the supernatant and inoculate it into 100 mL standard Luria–Bertani (LB) medium (composition: 10 g/L tryptone, 5 g/L yeast extract, 10 g/L NaCl, pH 7.0) standard Luria–Bertani medium. LB medium with 1%, 3%, 5%, 7%, 9%, 11% and 13% gradient salt concentration was used sequentially to screen the salt-resistant strains, then the 13% NaCl concentration was cultured by dilution coated plate method, and the single colonies with good growth were selected, purified repeatedly (streaked on LB agar plates 3 times), and then added with 50% glycerol (*v*/*v*, 1:1 ratio with bacterial suspension, total volume 500 μL) and put in −80 °C environment for conservation.

Bacterial DNA was extracted according to the Bacterial Genomic DNA Extraction Kit method (Tiangen Biotech; Beijing; China), and the products were sent to Sangon Biotech (Shanghai, China) for amplification and sequencing. The strain was identified by 16S rDNA analysis, and the obtained 16S rDNA gene sequence was submitted to the GenBank of NCBI for BLAST (https://www.ncbi.nlm.nih.gov/sra/PRJNA1221522, 1 July 2025) comparison and homology analysis with known sequences in the database under accession number PV390977.

### 2.2. Culture Conditions of the BSF-4 Strain

NaCl of 1–13% (*w*/*v*) was added to the medium, respectively, inoculated with 1% (*v*/*v*, 0.5 mL) bacterial suspension (OD_600_ ≈ 1.0) and cultured at 35 °C, 150 rpm, and the maximum and minimum concentration of NaCl and the optimal salt concentration of the strain were determined according to the light absorption value of the bacterial suspension at 600 nm under different NaCl concentrations.

**Figure 1:**
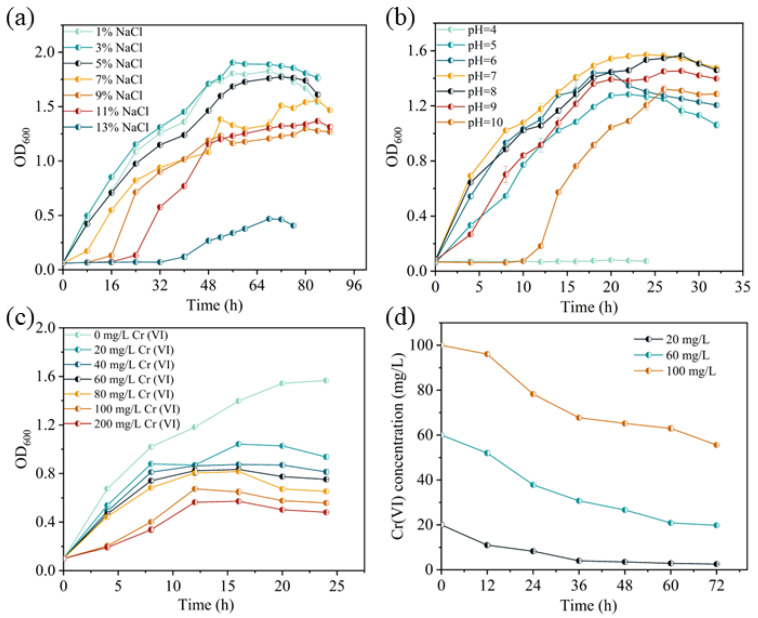
Effect of different factors on the growth and Cr(VI) reduction ability of strain *BSF-4*. (**a**) Effect of salinity. (**b**) Effect of pH. (**c**) Effect of initial Cr(VI) concentration. (**d**) Cr(VI) reduction capacity.

Liquid media adjusted to varying pH values (ranging from 5.0 to 10.0) were inoculated at a 1% inoculum ratio and incubated at 35 °C with shaking at 150 rpm to assess strain growth. Samples were collected at 4 h intervals for growth monitoring, and the assay was continued until the bacterial population entered the decline phase, as depicted in Figure 1. In subsequent experiments, bacteria were cultured at the optimum salt concentration and pH.

The growth of bacteria (OD_600_ nm) was determined every 4 h at different Cr(VI) concentrations (0, 20, 40, 60, 80, 100, and 200 mg/L) for Cr(VI) resistance test.

The *BSF-4* strain was inoculated into LB medium containing the initial Cr(VI) concentration of 0, 20, 60, and 100 mg/L, and cultured in an oscillating incubator at 35 °C and 170 rpm. The remaining hexavalent chromium concentration in the system was tested every 12 h.

In this investigation, potassium chromate (K_2_CrO_4_) was employed as the standard Cr(VI) source. Quantitative analysis of hexavalent chromium species was performed following the national standard method for water quality (GB 7467-1987) [31] utilizing the 1,5-diphenylcarbazide spectrophotometric quantification at 540 nm wavelength. Total chromium content was determined through a hot-block digestion system in accordance with GB 7466-1987 [32] specifications, where trivalent chromium Cr(III) was stoichiometrically oxidized to chromate ions via potassium permanganate (KMnO_4_) in strongly acidic conditions (pH < 2) at 100 °C. The Cr(III) concentration was subsequently calculated by subtracting the pre-oxidation Cr(VI) concentration from the total chromium measurement, with analytical quality control protocols including calibration curve verification and matrix spike recovery tests (85–115%) to ensure measurement accuracy.

### 2.3. Reduction Mechanism of Cr(VI) by BSF-4 Strain

#### 2.3.1. SEM-EDS Analysis [33]

The cell morphology under different Cr(VI) stresses (0, 20, 100 mg/L) was observed with field emission scanning electron microscopy (Sigma500, Zeiss, Oberkochen, Germany). The bacterial suspensions were inoculated into nutrient medium containing Cr(VI) free and 20 and 100 mg/L Cr(VI), respectively, cultured at 35 °C and 150 rpm to the logarithmic growth stage, centrifuged at 5000 rpm for 30 min, and the cell precipitates were rinsed with phosphate buffer for 2~3 times. Add 2.5% glutaraldehyde solution and mixed homogeneously, and the cells were fixed in refrigerator at 4 °C for 24 h, centrifuge again, eluted with 30, 50, 70, 90, and 100% anhydrous ethanol gradient, centrifuge discard supernatant, and then 500 μL of pure water was added to the cells and shaken well, drop 5 μL silicon wafers, and air dry naturally. After pretreatment, SEM observations were conducted in InLens mode at an accelerating voltage of 1.50 kV and a magnification of 10.00 k× (working distance was moderately adjusted for optimized imaging across samples).

#### 2.3.2. Chromium Speciation Analysis

To elucidate chromium fate, the prepared bacterial suspension was inoculated into the nutrient medium containing 20 mg/L Cr(VI) and cultured at 30 °C and 150 rpm for 48 h. Extracellular and intracellular Cr(VI) and total Cr concentrations were measured every 24 h, respectively. The treatment procedures were as follows [34]:

(1) Extracellular: Transfer the culture medium to be measured into a centrifuge tube, centrifuge at 5000 rpm for 30 min, take the supernatant, and determine Cr(VI) and total Cr concentration.
(2) Intracellular: The bacteria precipitated in the above step were repeatedly cleaned with phosphate buffer (pH = 7) for two times. After re-suspension with deionized water, the bacteria were broken by ultrasound in ice, centrifuged at 5000 rpm for 30 min, then the supernatant was taken to determine Cr(VI) and total Cr concentration.

#### 2.3.3. Cr(VI) Reduction Capacity of Bacterial Cell Components

Preparation of suspensions of different components of bacterial cells: extracellular metabolites, crude enzymes, and intact cells [34].

(1) Extracellular metabolites: The bacteria were inoculated into nutrient medium, incubated in oscillatory culture at 30 °C and 150 rpm until the logarithmic phase, centrifuged at 5000 rpm for 30 min, and the supernatant was taken. Extracellular metabolites were sampled after 0.22 μM.
(2) Intact cells: The cell precipitation in the previous step was repeatedly cleaned with phosphate buffer (pH = 7) for two to three times, divided into three parts, and one part was resuspended with deionized water, that is, to obtain an intact cell sample.
(3) Resting cells: Pelleted biomass underwent two cycles of phosphate-buffered saline (100 mM, pH = 7) washing and reconstitution, yielding metabolically quiescent cellular suspensions.
(4) Intracellular crude enzyme solution: A portion of the bacterial sediment obtained in step (2) was re-suspended with phosphate buffer, placed in an ice bath ultrasonic bacteria removal (100 W, 20 min), centrifuged at 5000 rpm for 30 min, and the supernatant was taken as the crude enzyme extract sample.

The rest cell suspension was added with 20 mg/L Cr(VI), and the other components of the suspension were inoculated into the nutrient medium containing 20 mg/L Cr(VI), and cultured at 30 °C and 150 rpm. The Cr(VI) concentration was measured by sampling at intervals. The experiment was set up for three replicates.

#### 2.3.4. XPS Analysis

The *BSF-4* strain was cultured to logarithmic phase, and an appropriate amount of bacterial liquid was transferred to a centrifuge tube and centrifuged at 8000 rpm at room temperature for 10 min. After separating the supernatant from the bacterial precipitation, anhydrous ethanol was added to the supernatant and placed in a refrigerator at 4 °C overnight. The supernatant was removed by centrifugation the next day, leaving the extracellular polymer precipitation. The bacteria precipitates were cleaned twice with deionized water, and then the washed bacteria and extracellular polymer components were put into a freeze-dryer to lyophilize for 48 h, and then the freeze-dried strain powder was placed in an XPS scanning table for scanning. X-ray photoelectron spectroscopy (XPS) was conducted on lyophilized biomass using an ESCALAB Xi+ spectrometer (Thermo Fisher Scientific, Waltham, MA, USA) with monochromatic Al Kα radiation (300 W). Surface chromium speciation was deconvoluted via Avantage Software (version 5.9931), employing Cr-free biomass as spectral reference.

### 2.4. Transcriptomics Analysis

Activated *BSF-4* strain was cultured and inoculated into the medium containing 20 mg/L Cr(VI) in logarithmic stage, and the bacterial samples at 12 h, 24 h, 36 h and 48 h moments were taken and labeled as W1, W2, W3 and W4, respectively. In three parallel experiment groups, and one control group W0 which without Cr(VI), a total of 15 samples were sent to novogene Ltd. (Beijing, China) to extract prokaryotic *RNA* for high-throughput transcriptome sequencing (sequencing results are provided in Text S1). The *RNA* samples were subjected to strict quality control by means of Agilent 2100 bioanalyzer (Santa Clara, CA, USA), and after the extracted *RNA* was detected to be qualified, the enriched *mRNA* of ribosomal *RNA* (*rRNA*) was removed from the total *RNA* with a probe.

Subsequently, the obtained *mRNA* was randomly interrupted into short fragments by adding the fragment buffer, and then the libraries were constructed according to the strand-specific library construction method. After the library inspection was qualified, different libraries were pooled according to effective concentration and target downstream data volume, and then sequenced by Illumina. The KEGG pathway enrichment analysis of differential gene sets was performed using clusterProfiler software (version v3.8.1). Quantitative real-time PCR was used to verify the transcriptional changes in Cr(VI) tolerance and reduction-related genes (Figure S4).

## 3. Results and Discussion

### 3.1. Identification and Characterization of Strain BSF-4

The strain *BSF-4* isolated from saline-alkali soil was identified as *Bacillus safensis* and Gram-positive bacteria by 16S rDNA sequencing. According to the measurement of the growth curve under the salt concentration gradient, with the increase in NaCl concentration, the growth rate of the absorbance value of the bacterial solution gradually slowed down. When the salt concentration was as high as 13%, the bacterial growth and reproduction were severely inhibited (Figure 1a). It proved that the bacterium is moderately halotolerant and can survive within the salt concentration range of 1% to 11%. At the same time, its most suitable salt concentration for growth is 3%. It was cultured under optimal salt concentration conditions, and its optimal growth pH was 7 through an alkali resistance test. Excessive acidity or alkalinity would inhibit the growth of the strain. When the pH was 4, the bacteria showed no signs of growth after 24 h of continuous monitoring, and it was considered that the bacteria did not grow (Figure 1b). The Cr(VI) resistance test of bacteria cultured under optimal salt concentration and pH showed that strain *BSF-4* was able to tolerate at least 200 mg/L of Cr(VI), but with the increase in Cr(VI) concentration, cell growth was progressively inhibited (Figure 1c). Further research on the removal efficiency of Cr(VI) shows that when the initial concentration of *BSF-4* is 20, 60, and 100 mg/L, the reduction efficiency of Cr(VI) within 72 h is 89.15%, 66.95%, and 44.42%, respectively (Figure 1d).

**Figure 2:**
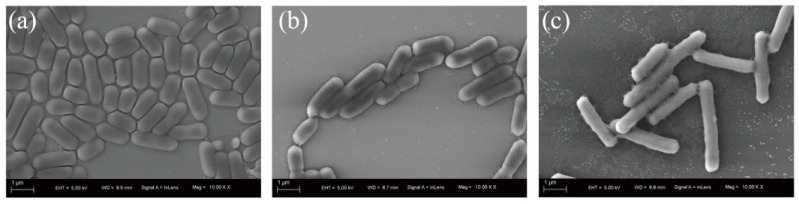
Scanning Electron Microscope (SEM) micrographs of cell morphology after incubation for 24 h with different Cr(VI) concentrations. (**a**) 0 mg/L Cr(VI); (**b**) 20 mg/L Cr(VI); and (**c**) 100 mg/L Cr(VI).

**Figure 3:**
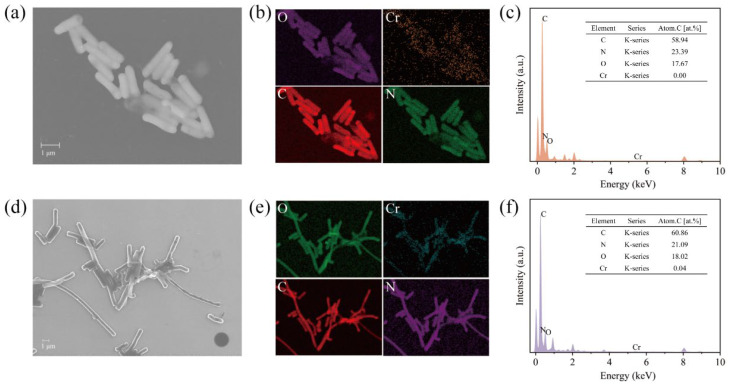
Transmission Electron Microscope-Energy Dispersive Spectroscopy (TEM-EDS) spectra of *BSF-4* under Cr(VI) stress. (**a**) TEM of 20 mg/L Cr(VI); (**b**) Mapping of 20 mg/L Cr(VI); (**c**) EDS of 20 mg/L Cr(VI); (**d**) TEM of 100 mg/L Cr(VI); (**e**) Mapping of 100 mg/L Cr(VI); and (**f**) EDS of 100 mg/L Cr(VI).

This strain showed an amazing effect of degrading Cr(VI), but its degradation mechanism was not clear. Therefore, we first observed the morphological changes in *BSF-4* strain cells under Cr(VI) stress by scanning electron microscopy. Compared with the normal condition, the bacterial morphology did not change significantly under the treatment of 20 mg/L Cr(VI) (Figure 2b), and the cells were significantly elongated after 100 mg/L Cr(VI) stress (Figure 2c). The surface of the bacterium remained smooth, and no adsorption mass was found at all times. This observed cellular elongation may represent a stress-responsive strategy to reduce the surface area-to-volume ratio, thereby potentially minimizing Cr(VI) permeation. Microscopic examinations further revealed slight local indentations in the cell wall; however, the overall cellular morphology remained intact without obvious surface damage. Concomitant with the significant increase in cell length, intercellular aggregation was enhanced. These morphological adaptations collectively suggest that the strain may employ strategies such as biofilm formation and morphological modification to resist the toxicity of high-concentration Cr(VI) [35,36]. Combined with TEM-Mapping analysis, no attached chromium element was found on the surface of bacteria, which proves that the reduction in Cr(VI) was not due to the adsorption of bacteria (Figure 3c,f).

### 3.2. Reduction Mechanism of Cr(VI) by Strain BSF-4

On the basis of excluding the adsorption of Cr(VI) by strain *BSF-4*, the reduction in Cr(VI) was explored. In order to detect the existence of Cr(VI) reduction products of *Bacillus* sp. In *BSF-4*, the distribution location of Cr(VI) reduction products was analyzed. Intracellular and extracellular Cr(VI) and total Cr contents were detected, respectively. The content of Cr(III) in the reduction product was the difference between the total Cr content and the Cr(VI) content. After being cultured in the medium with a hexavalent chromium concentration of 20 mg/L, the extracellular residual Cr(VI) concentration was 7.933 mg/L, and the total chromium concentration was 13.575 mg/L after 48 h. The intracellular Cr(VI) concentration was 0.31 mg/L, and the total chromium concentration was 1.15 mg/L (Table 1). This part of the experiment indicates that part of Cr(VI) enters the cell interior and is reduced, and the reduction products mainly exist in the supernatant. After the reaction, the total amount of intracellular and extracellular chromium was not much different from the initial hexavalent chromium, which further indicated that the adsorption amount of Cr(VI) was very small, but rather was converted into other valence states.

**Figure 4:**
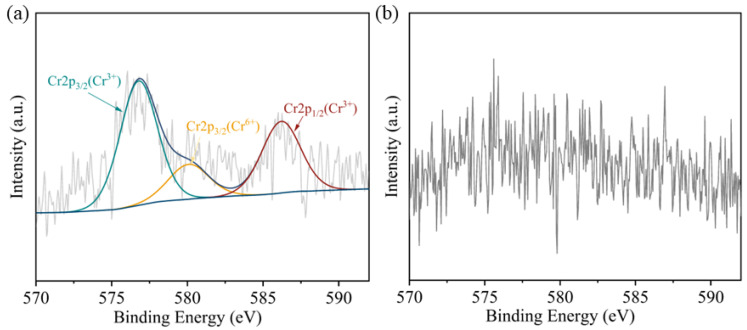
X-ray photoelectron spectroscopy analysis of chromium valence states. (**a**) XPS of extracellular polymers. (**b**) XPS of *BSF-4* bacteriophage cell.

It is generally accepted that the microbial Cr(VI) reduction process will form insoluble Cr(III) precipitation, such as Cr(OH)_3_ and Cr_2_O_3_ [37]. However, in recent years, some studies have shown that the reduction products of Cr(VI) exist in soluble forms [38]. Like the soluble Cr(III)-NAD^+^ complex [39], Cr(III)-EPS complex [40]. The valence state and location of reduced chromium were further determined by XPS, and Figure 4a showed that two distinct peaks were obtained from the extracellular polymer sample of strain *BSF-4*. The peaks were in the range of 585.0–588.0 eV (Cr 2p1/2) and 576.0–578.0 eV (Cr 2p3/2), respectively, consistent with the spectra of Cr^3+^ according to previous literature references [41,42], 580 eV is an under-reduced Cr^6+^ [43], Figure 4b shows the bacterial precipitation scanned, and no chromium peak was found. This indicates that Cr(III) did not form insoluble substances adsorbed on the surface of bacteria, and it can be assumed that Cr(III) exists mainly in the organic complex state in solution [44]. It was again demonstrated that the reduction in Cr(VI) by this strain of *Bacillus safensis BSF-4* was mainly due to reduction rather than adsorption.

**Figure 5:**
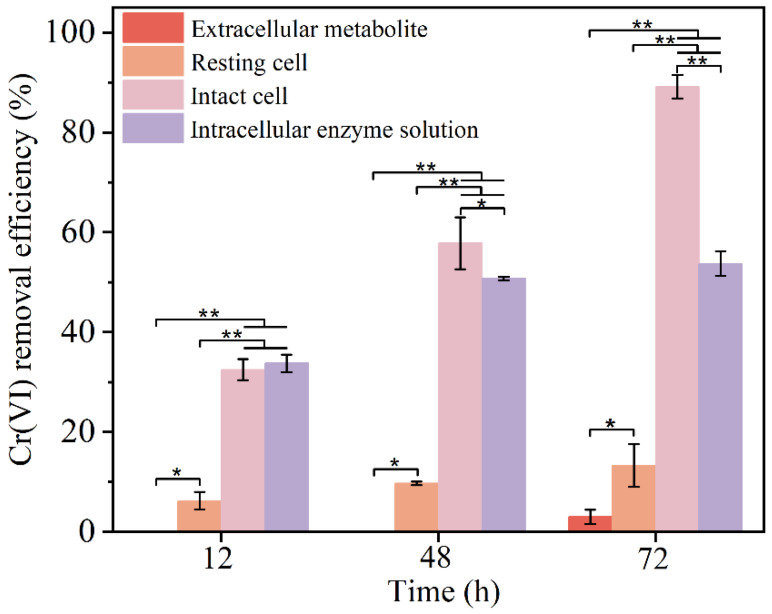
Degradation efficiency of Cr(VI) by cellular components.* *p* < 0.05, ** *p* < 0.01.

In addition, the basic conditions of growth and metabolism of *Bacillus safensis BSF-4* and its reduction characteristics in chromium-containing medium were investigated previously. In order to eliminate the interference of the bacterial metabolism on the reduction in Cr(VI), an experiment was designed with resting cells [34]. So that to further study the mechanism of Cr(VI) reduction by strain *BSF-4*, each cellular component was separated and its Cr(VI) reduction effect was determined separately. It was found that the reduction ability of Cr(VI) was different among cell components, and the reduction effect of Cr(VI) on intact cells, resting cells, extracellular secretions, and crude enzymes was shown in Figure 5.

With the increase in time, the reduction efficiency of each component increased. After 24 h, the reduction efficiency of extracellular components was not detected, and the reduction rate of intact cells was 32.5%, that of crude enzymes and resting cells was 33.75% and 6.25%, respectively. At 48 h, the reduction efficiency of each component (extracellular component, intact cell, crude enzyme, and resting cell) was 0.025%, 57.83%, 50.75% and 9.75%, respectively. The reduction efficiency of Cr(VI) of each component was 3%, 89.15%, 53.75% and 13.275% at 72 h, in order of magnitude. Overall, intact cells had the strongest reduction effect, followed by crude enzymes; resting cells and extracellular cells had poorer reducing ability. This may be due to the fact that some reducing substances, such as reductases and cytochromes secreted by cells, enter the supernatant and carry out a reduction effect outside the cell [45] to resist the stress of Cr(VI). The reducing ability of resting cells to Cr(VI) is much lower than that of intact cells, which proves that Cr(VI) reduction may be related to the metabolism and reproduction of cells. Due to the damage of reducing bacteria cells, they cannot undergo outgrowth and metabolism to produce a large amount of reductase and other substances, and the reducing ability of each cell component is greatly limited.

In summary, the reduction site of Cr(VI) by this bacterium occurred outside the cell, but the extracellular supernatant had almost no reducing capacity for Cr(VI) ions. Therefore, it was inferred that there existed some pathway in the bacterium to secrete the relevant enzyme proteins from the intracellular to complete the reduction metabolism of chromium ions through transmembrane transport. Thus, we further delved into the mechanism of the Cr(VI) reduction by the transcriptomics analysis.

### 3.3. Identification of Genes Involved in Cr(VI) Reduction

**Figure 6:**
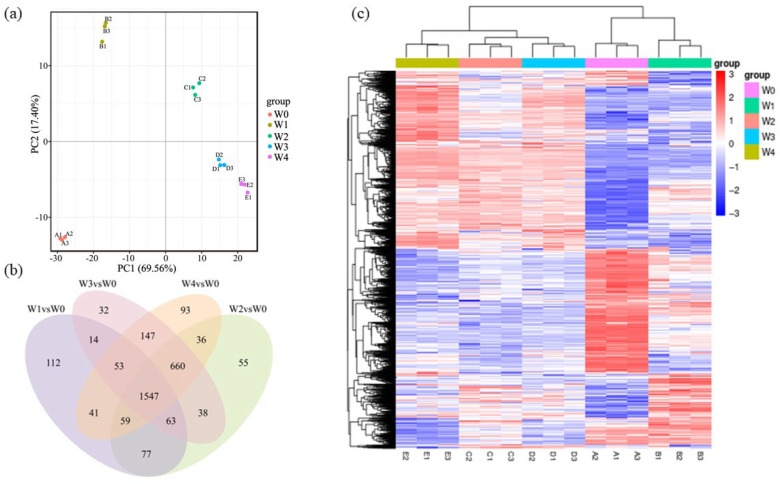
Results of transcriptomic data analysis. (**a**) Principal component analysis of gene expression. (The horizontal coordinate of the graph is the first principal component, and the vertical coordinate is the second principal component). (**b**) Venn diagram of differential genes in different samples. (**c**) Heat map of differential gene clustering. (Horizontal coordinates are sample names, and vertical coordinates are the normalized differential gene FPKM value).

After quality control, the original transcriptome data obtained clean bases ≥ 1.0 G, GC content (%) ≥ 42.65%, the sample compared with the reference genome, the total mapping rate was >92%, Q20(%) content was >97%, Q30(%) content was >93.63% (Table S1). The transcriptome data obtained are of high quality and can be used for subsequent analysis. PCA analysis of gene expression values (FPKM) of all samples (Figure 6a) shows high variability of samples between groups and good parallel correlation of samples within groups. Figure 6b compares the overlap of differential genes among the experimental combinations. The four groups shared 1547 differential genes, and the W1, W2, W3, and W4 groups had unique 112, 32, 93, and 55 differential genes, respectively. Figure 6c Cluster analysis of gene expression values uses mainstream hierarchical clustering to homogenize expression data rows. Genes or samples with similar expression patterns are clustered together as shown in the figure, with red indicating high gene expression and blue indicating low gene expression. The transcriptomic data of all experimental groups have been uploaded to the NCBI database, with the accession number PRJNA1221522.

Four groups of samples were screened for significantly different expression levels of genes in different states (DESeq2 padj <= 0.05 |log2FoldChange| >= 0.0). In the process of Cr(VI) reduction by the *BSF-4* strain, the genes encoding cytochrome C proteins, mainly cytochrome c oxidase subunit IV (*ctaF*), cytochrome c oxidase subunit III (*ctaE*), and cytochrome c oxidase subunit II (*coxB*), are significantly up-regulated. At 12 h, 24 h, 36 h, and 48 h, the *ctaF* gene was up-regulated 15.41 times, 23.84 times, 26.45 times, and 23.86 times, *ctaE* was up-regulated more than 26 times, and the *coxB* gene was up-regulated 11.41 times, 21.95 times, 25.70 times and 22.54 times, respectively, to participate in the oxidative phosphorylation pathway. In this process of electron transfer, Cr(VI) acts as an electron acceptor, and cytochrome proteins act as an electron donor. Under the catalysis of intracellular, cell membrane, or extracellular reductase, the transformation of Cr(VI) to Cr(III) is realized. Previous studies have found that cytochrome C proteins play an important role in Cr(VI) reduction by transporting electron donors to the extracellular compartment to complete Cr(VI) reduction [46,47]. The *BSF-4* strain is a kind of bacterium with good resistance to the toxic effects of Cr(VI), and one of the effective regulatory ways to resist the toxic effects of Cr(VI) is to reduce the uptake of Cr(VI) and prevent Cr(VI) from entering bacterial cells. Similar to the sulfate uptake pathway, CrO_4_^2−^ and SO_4_^2−^ have similar molecular structures; it can easily pass through cell membranes via the SO_4_^2−^ transport pathway [48], with the help of non-specific anionic (SO_4_^2−^, PO_4_^3−^) carriers [49]. We found that genes related to sulfate transport, such as adenylyl-sulfate kinase (*cysC*), adenosine 5′-phosphosulfate reductase (*cysH*), and sulfite reductase [NADPH] flavoprotein alpha-component (*cysJ*), were significantly down-regulated, and the *cysC* gene was down-regulated 2.11 times, 7.07 times, and 6.00 times at 12 h, 24 h, 36 h, and 48 h, respectively, and the phosphate uptake pathway was impeded. As a result, the Cr(VI) entering the cell through the sulfate channel in the *BSF-4* strain was reduced, which in turn resists the high concentration of Cr stress in the external environment [48].

Genes related to ABC transport, such as dipeptide transport system permease protein Dppc (*dppC*) and Dppb (*dppB*), nickel ABC transporter substrate-binding protein Nikd (*nikD*) and Nika (*nikA*) genes encoding polypeptide transport complexes, glutamine-binding periplasmic protein Glnh (*glnH*) and Glnp (*glnP*) encoding glutamine, were significantly up-regulated, so the ABC transporter protein system is also involved in Cr(VI) resistance [50]. In addition, the formaldehyde dehydrogenase gene (*fdhA*), which is related to glutathione, was up-regulated 11.03 times, 22.24 times, 15.37 times, and 11.58 times at 12 h, 24 h, 36 h, and 48 h, respectively, which was attributed to the fact that ascorbic acid is usually absent from bacterial cells, and the reducing glutathione can slowly reduce chromate [51].

During the process of chromium hexavalent entering cells, reactive oxygen species (ROS) can be formed to interact with proteins and nucleic acids, resulting in harmful effects on cells [52,53]. At this time, Cr(VI) stress will stimulate the bacterial stress response (SOS response) and DNA repair, and cells will improve the synthesis of intracellular catalase, superoxide dismutase (SOD), and glutathione S-transferase to counteract the oxidative stress damage to cells [54]. Superoxide dismutase (SOD) is an antioxidant metal enzyme present in living organisms, and the expression of superoxide dismutase Fe (*sodF*) gene encoding superoxide dismutase in the *BSF-4* strain was up-regulated 8.70 times, 41.32 times, 106.41 times, and 195.26 times at 12 h, 24 h, 36 h, and 48 h, respectively. In addition, since intracellular Cr(VI) can cause DNA breakage, *BSF-4* strain also regulates and activates DNA helicases, ATP-dependent DNA helicase RecG (*recG*), and holliday junction branch migration DNA helicase RuvB (*ruvB*), which are involved in the DNA repair mechanism to repair damaged DNA [55,56], which also helps the *BSF-4* strain resist Cr(VI) stress. At the same time, Cr(VI) stress will stimulate the quorum-sensing mechanism of bacteria [57]. Bacterial quorum sensing is the ability to detect and respond to cell population density through gene regulation and is a form of communication that promotes interactions between bacterial cells in a population [58]. In the *BSF-4* strain, the transfer genes encoding membrane protein, mainly ABC transporter permease (*appB*), were significantly up-regulated 40.40 times, 83.37 times, 36.10 times, and 57.73 times at 12 h, 24 h, 36 h, and 48 h, respectively. These genes play an important role in quorum sensing mechanism; therefore, it is inferred that the *BSF-4* strain performs Cr(VI) reduction by secreting intracellular reductive proteins into the extracellular.

**Figure 7:**
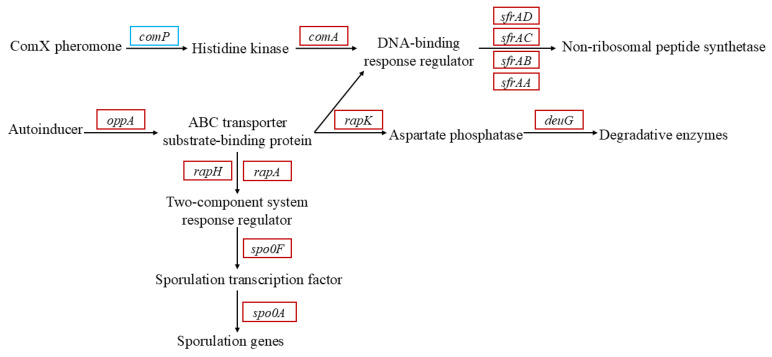
KEGG focuses on the relevant pathways of quorum sensing. Red solid boxes represent up-regulated genes, while blue solid boxes represent down-regulated genes.

As shown in Figure 7, in the whole expression system, response regulator aspartate phosphatase K (*rapK*), *rapA* and *rapH* genes act as response regulators to sense the signal of ABC transport-binding protein, and significantly up-regulated gene expression, which was transmitted to the *degU* gene (encoding bacterial regulatory proteins) in response to the reception of regulatory factors, and increased the expression of the product-degrading enzyme to resist the stress of hexavalent chromium toxicity on cells. Moreover, a signal is transmitted to the sporulation initiation phosphotransferase F (*Spo0F*) response regulator, and stage 0 sporulation protein A (*Spo0A*), a spore initiator, receives the signal, and a large number of spores are produced to ensure the survival of bacteria in the extreme environment Cr(VI). Simultaneous *sfrA* family genes are up-regulated to generate bacterial surface activators to cope with chromium stress. This process is mediated by autoinducers, and substantial upregulation of amino acid permease genes was observed, correlating with increased substrate-level phosphorylation capacity required for quorum-sensing autoinducer biosynthesis and transmembrane transport [59]. The genes mentioned above are listed in Table S3.

## 4. Conclusions

In this study, a halotolerant bacterium, *Bacillus safensis BSF-4*, was successfully isolated from saline-alkali soil, with systematic experiments revealing several key findings: under optimal culture conditions (35 °C, 150 rpm, pH 7.0–8.0), the strain exhibited remarkable Cr(VI) reduction capacity, achieving an 89.15% reduction efficiency for 100 mg/L Cr(VI) within 72 h, and this efficiency remained stable even under high-salt stress, highlighting its adaptability to harsh environments. Mechanistically, physiological and molecular assays indicated that *BSF-4* mediates Cr(VI) reduction through multiple pathways—electron donor transfer via cytochrome C proteins, transmembrane transport of Cr(VI) via ABC transporters, and regulatory coordination through quorum sensing, while resisting Cr(VI) toxicity by activating ROS detoxification systems (e.g., enhanced superoxide dismutase activity) and DNA repair mechanisms, validated by elevated expression of related functional genes. This is the first report documenting *Bacillus safensis* as a strain with both exceptional salt tolerance and high-efficiency Cr(VI) reduction, bridging the gap in understanding Cr(VI) biotransformation in saline-alkali contaminated environments. This study provides a theoretical basis for detoxification and transformation of Cr(VI) in contaminated environments, emphasizing the strain’s ability to convert toxic Cr(VI) to low-toxicity Cr(III), as well as offering a new idea for the study of Cr(VI) reduction mechanism, which supplies an important scientific basis and technical support for future remediation work.

Subsequent research will focus on in-depth functional characterization of key genes involved in Cr(VI) reduction, including cytochrome C-encoding genes and ABC transporter-related genes, through gene editing techniques (e.g., overexpression or site-directed mutagenesis). This will clarify their specific regulatory nodes in the electron transport chain and facilitate the construction of engineered strains with enhanced Cr(VI) reduction efficiency for practical bioremediation. To further explore the interaction between the bacterial system and Cr(VI) in the medium, we will conduct Visual Minteq modeling to analyze the bioavailability of chromium. This analysis will help us understand how the speciation and bioavailability of chromium influence bacterial reduction processes, bridging the gap in understanding the environmental behavior of Cr(VI) in our study system. Given that Cr(VI) toxicity primarily arises from its oxidizing property, future experiments will integrate measurements of oxidative stress markers (e.g., ROS levels, SOD/CAT activities) and systematic analyses of extracellular polymeric substances (EPS) components (e.g., total sugars, proteins, functional groups) to comprehensively elucidate the reduction mechanism and strengthen the mechanistic completeness of this study.

## Figures and Tables

**Table 1:** Extracellular and intracellular residual Cr(VI) and total Cr concentrations at 48 h in the system.

Location	Initial Cr(VI) (mg/L)	Residual Cr(VI) (mg/L)	Total Cr (mg/L)
extracellular	19.25	7.93	13.58
intracellular	0.00	0.31	1.15

## Data Availability

The transcriptomics data have been deposited in the Sequence Read Archive (SRA) database under the accession number PRJNA1221522. The complete datasets can be accessed here: https://dataview.ncbi.nlm.nih.gov/object/PRJNA1221522?reviewer=6fi9qajj98qjt2avmi5uu604l3 (accessed on 1 July 2025).

## Supplementary Materials

The following supporting information can be downloaded at: https://www.mdpi.com/article/10.3390/microorganisms13081961/s1.
