# Supplementary material for: Extracellular Cr(VI) Reduction by the Salt-Tolerant Strain Bacillus safensis BSF-4
Source: Microorganisms. 2025 Aug 21;13(8):1961. doi: 10.3390/microorganisms13081961 (PMC12388424; doi:10.3390/microorganisms13081961)
Supplement: Supplementary file 1 [file microorganisms-13-01961-s001.zip › microorganisms-3747365-supplementary.pdf]

SUPPORTING INFORMATION: Extracellular Cr(VI) Reduction by the Salt-Tolerant Strain *Bacillus safensis BSF-4*

Yilan Liu [1], Weiping Yu [2], Tianying Nie [1], Lu Wang [1,*] and Yusheng Niu [1,2,*]

[1] Institute of Biomedical Engineering, College of Life Sciences, Qingdao University, Qingdao 266071, China; liuyilan2022@163.com (Y.L.); ying18854709906@163.com (T.N.)

[2] Research Institute of Modern Agricultural Industry Innovation in Yellow River Delta Saline-Alkali Land, Dongying Vocational College, Dongying 257029, China; adongzi@126.com

* Correspondence: lwang@qdu.edu.cn (L.W.); nys@qdu.edu.cn (Y.N.)

Text S1. Sequencing results of BSF-4 16S rDNA

TGGGGAGGCTATACATGCAGTCGAGCGGACAGAAGGGAGCTTGCTCCCGG

ATGTTAGCGGCGGACGGGTGAGTAACACGTGGGTAACCTGCCTGTAAGAC

TGGGATAACTCCGGGAAACCGGAGCTAATACCGGATAGTTCCTTGAACCGC

ATGGTTCAAGGATGAAAGACGGTTTCGGCTGTCACTTACAGATGGACCCGC

GGCGCATTAGCTAGTTGGTGGGGTAATGGCTCACCAAGGCGACGATGCGTA

GCCGACCTGAGAGGGTGATCGGCCACACTGGGACTGAGACACGGCCCAG

ACTCCTACGGGAGGCAGCAGTAGGGAATCTTCCGCAATGGACGAAAGTCT

GACGGAGCAACGCCGCGTGAGTGATGAAGGTTTTCGGATCGTAAAGCTCT

GTTGTTAGGGAAGAACAAGTGCGAGAGTAACTGCTCGCACCTTGACGGTA

CCTAACCAGAAAGCCACGGCTAACTACGTGCCAGCAGCCGCGGTAATACGT

AGGTGGCAAGCGTTGTCCGGAATTATTGGGCGTAAAGGGCTCGCAGGCGG

TTTCTTAAGTCTGATGTGAAAGCCCCCGGCTCAACCGGGGAGGGTCATTGG

AAACTGGGAAACTTGAGTGCAGAAGAGGAGAGTGGAATTCCACGTGTAGC

GGTGAAATGCGTAGAGATGTGGAGGAACACCAGTGGCGAAGGCGACTCTC

TGGTCTGTAACTGACGCTGAGGAGCGAAAGCGTGGGGAGCGAACAGGATT

AGATACCCTGGTAGTCCACGCCGTAAACGATGAGTGCTAAGTGTTAGGGGG

TTTCCGCCCCTTAGTGCTGCAGCTAACGCATTAAGCACTCCGCCTGGGGAG

TACGGTCGCAAGACTGAAACTCAAAGGAATTGACGGGGGCCCGCACAAGC

GGTGGAGCATGTGGTTTAATTCGAAGCAACGCGAAGAACCTTACCAGGTCT

TGACATCCTCTGACAACCCTAGAGATAGGGCTTTCCCTTCGGGGACAGAGT

GACAGGTGGTGCATGGTTGTCGTCAGCTCGTGTCGTGAGATGTTGGGTTAA

GTCCCGCAACGAGCGCAACCCTTGATCTTAGTTGCCAGCATTCAGTTGGGC

ACTCTAAGGTGACTGCCGGTGACAAACCGGAGGAAGGTGGGGATGACGTC

AAATCATCATGCCCCTTATGACCTGGGCTACACACGTGCTACAATGGACAG

AACAAAGGGCTGCAAGACCGCAAGGTTTAGCCAATCCCATAAATCTGTTCT

CAGTTCGGATCGCAGTCTGCAACTCGACTGCGTGAAGCTGGAATCGCTAGT

AATCGCGGATCAGCATGCCGCGGTGAATACGTTCCCGGGCCTTGTACACAC

CGCCCGTCACACCACGAGAGTTTGCAACACCCGAAGTCGGTGAGGTAACC

TTTATGGAGCCAGCCGCCGAAAGGACCT

Table S1. Summary of sample sequencing data quality

Sample name	Raw reads	Clean reads	Raw bases	Clean bases	Error rate (%)	Q20(%)	Q30(%)	GC content(%)
A1	9269992	9013236	1.4G	1.4G	0.01	98.98	96.89	43.04
A2	8347162	8083660	1.3G	1.2G	0.01	98.9	96.69	43.22
A3	6923056	6697524	1.0G	1.0G	0.01	98.77	96.36	43.06
B1	7551924	7387738	1.1G	1.1G	0.01	98.83	96.48	42.65
B2	7769116	7570904	1.2G	1.1G	0.01	98.21	94.6	42.93
B3	7801982	7644940	1.2G	1.1G	0.01	98.04	94.11	42.71
C1	7840916	7630750	1.2G	1.1G	0.01	98.13	94.46	42.99
C2	7788612	7309446	1.2G	1.1G	0.01	97.92	93.95	43.13
C3	7636978	7406650	1.1G	1.1G	0.01	98.88	96.67	42.78
D1	7690628	7486850	1.2G	1.1G	0.01	98.11	94.4	43.51
D2	7556180	6953894	1.1G	1.0G	0.01	97.83	93.63	43.22
D3	7602922	7353044	1.1G	1.1G	0.01	98.74	96.24	43.24
E1	7273464	6653036	1.1G	1.0G	0.01	98.7	96.24	43.43
E2	7619270	7313194	1.1G	1.1G	0.01	98.91	96.72	43.41
E3	7742902	7527778	1.2G	1.1G	0.01	98.89	96.69	43.37

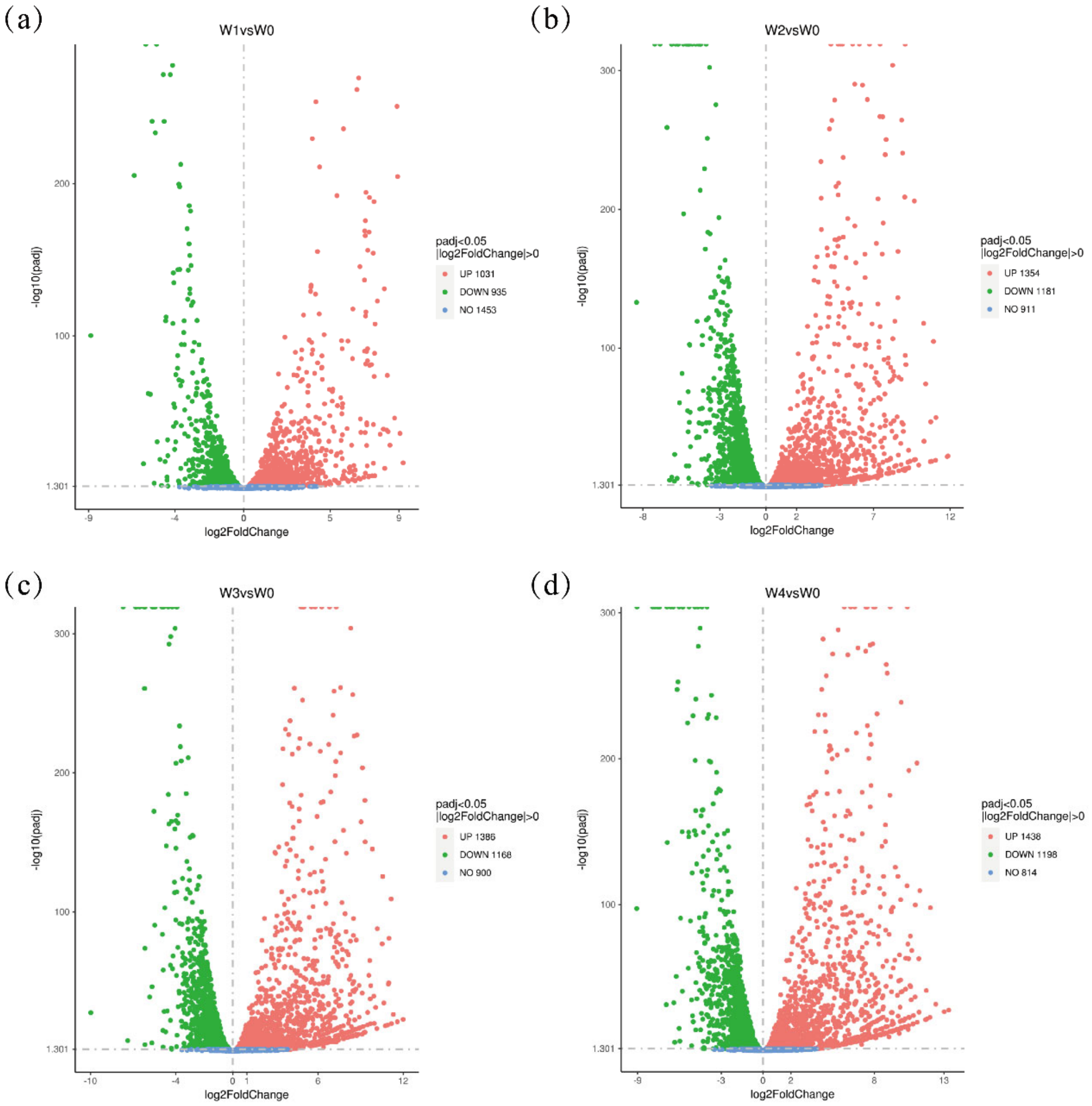

Figure S1. Variance analysis volcano plot. (a) After 12h of hexavalent chromium chromium stress, there were 1031 up-regulated genes and 935 down-regulated genes compared to controls. (b) After 24h of hexavalent chromium chromium stress, there were 1354 up-regulated genes and 1181 down-regulated genes compared to controls. (c) After 36h of hexavalent chromium chromium stress, there were 1386 up-regulated genes and 1168 down-regulated genes compared to controls. (d) After 48h of hexavalent chromium chromium stress, there were 1438 up-regulated genes and 1198 down-regulated genes compared to controls.

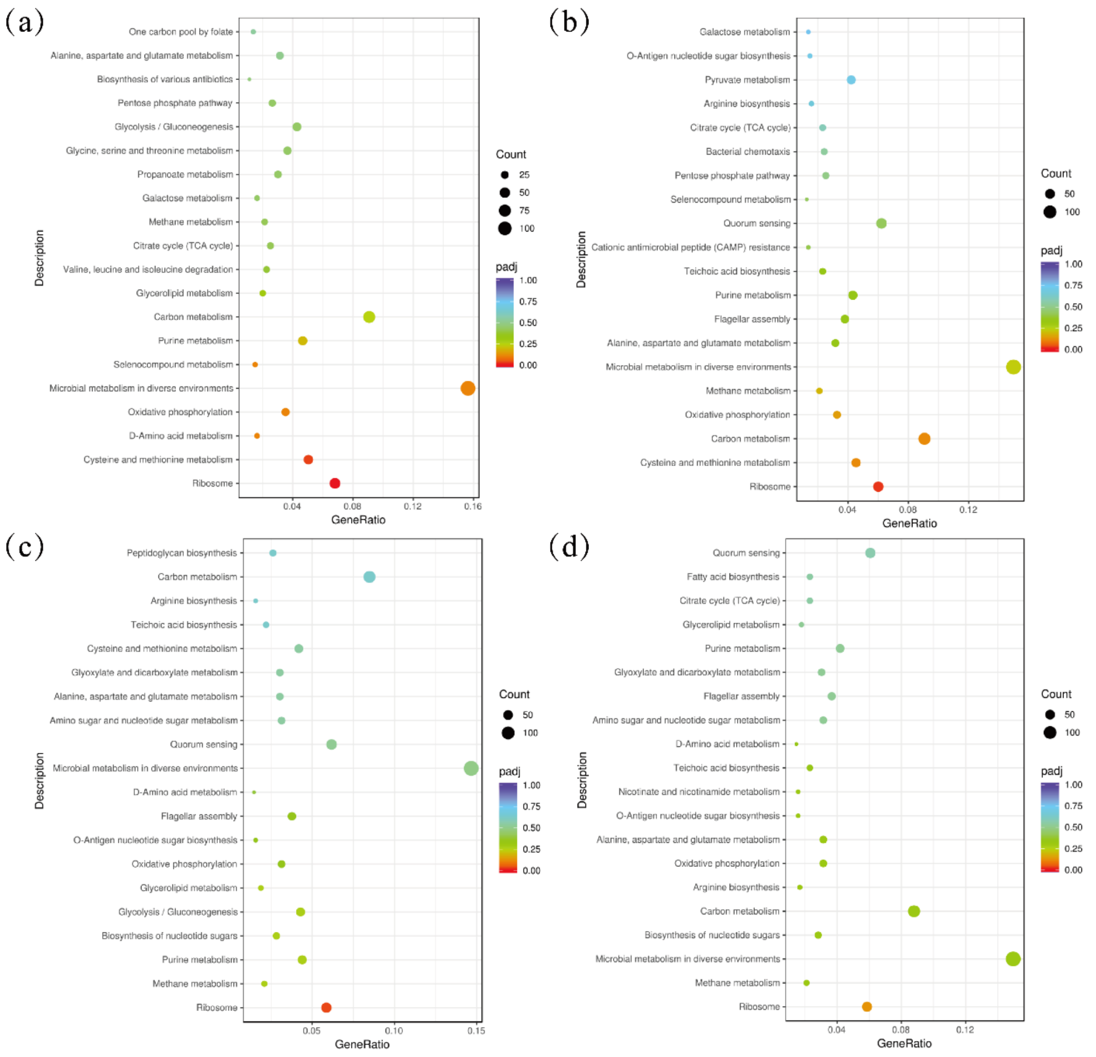

Figure S2. KEGG enrichment analysis graph Top20. The horizontal coordinate of the figure is the ratio of the number of differential genes annotated to the KEGG pathway to the total number of differential genes, and the vertical coordinate is the KEGG pathway. The scatterplot is shown with different colours and the size of the dots, and the colours show a gradual change from purple to red. The redder the colours are, the more significant the enrichment is, and the bigger the dots are, the more genes are enriched on the enriched genes (padj < 0.05).

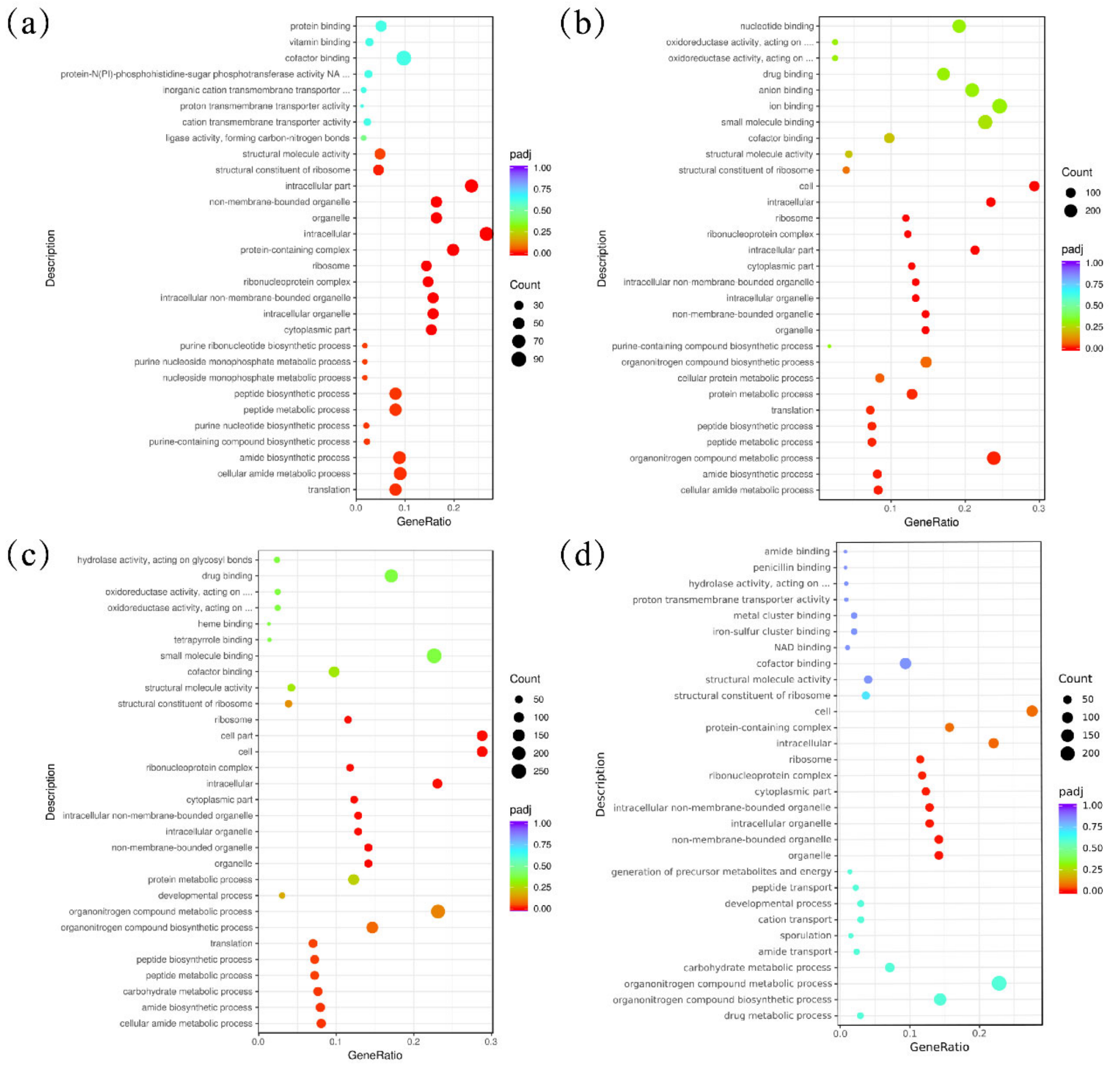

Figure S3. GO enrichment analysis graph. Horizontal coordinate is the ratio of genes annotated to the function to the total number of differential genes, vertical coordinate GO Term, Scatterplot is shown in different colors and the size of the dots, the color is gradient from purple to red, the redder the colour, the more significant the enrichment; the bigger the dots, the more genes on the enrichment (padj < 0.05).

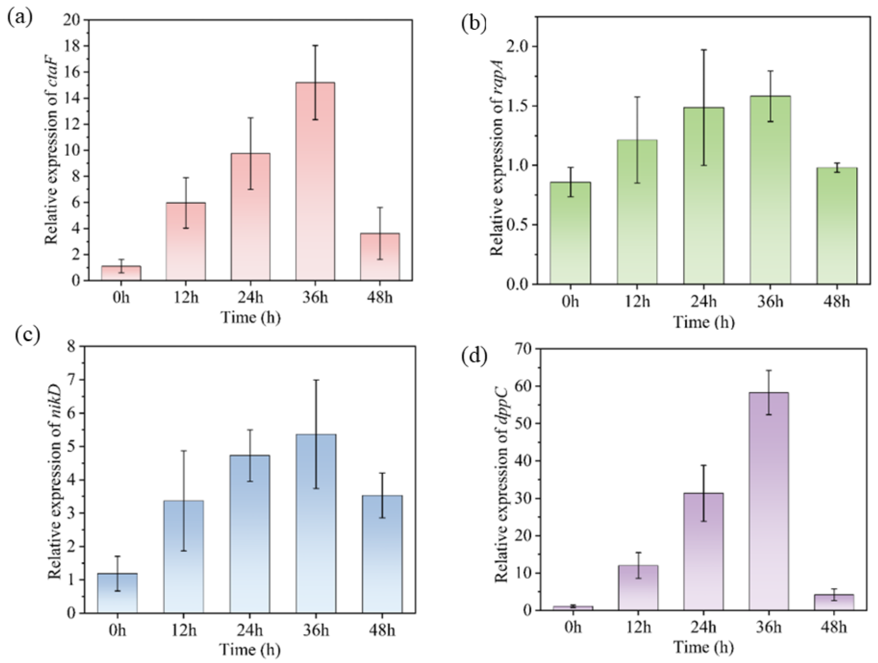

Figure S4. Relative gene expression levels of *ctaF*、*rapA*、*nikD* and *dppC* at 20 mg/L Cr(VI).

Table S2. qPCR Primer Sequences

Primer	Sequence	Gene
rpoB-S	AATGCCGAGGAA	*rpoB*
rpoB-A	GGAAATCACCCATAA	
ctaF-S	AATGCCGAGGAA	*ctaF*
caF-A	CTGCGAACCAAT	
rapA-S	GCTTATTACCGCTCAG	*rapA*
rapA-A	AAGTGGGTTTGTTTCA	
nikD-S	GCTGCGGGAAAACAGTAACAAG	*nikD*
nikD-A	TTGCTGCTGCTGCTGCTG	
dppC-S	CTGCTGCCGAATACGAT	*dppC*
dppC-A	GAATACCTAAACCGAGAAAG	

Table S3. Some key genes resistant to Cr (VI) str

Gene name	log2FoldChange of W1	log2FoldChange of W2	log2FoldChange of W3	log2FoldChange of W4	Pvalue of W1	Pvalue of W2	Pvalue of W3	Pvalue of W4	Gene description
ctaF	3.945795377	4.575811972	4.72557492	4.576283057	2.22E-90	1.72499721489794E-180	2.00189465402884E-227	2.82388213195396E-193	cytochrome c oxidase subunit IV
ctaE	4.104622417	4.705763729	4.916416388	4.791212316	1.01464552468312E-77	5.80E-213	3.73574165975429E-255	2.4771449951837E-211	cytochrome c oxidase subunit III
coxB	3.511776423	4.455916153	4.683507781	4.494609594	4.9372773072477E-38	4.43344803401772E-150	1.03844061738138E-167	3.10944362221765E-221	cytochrome c oxidase subunit II
cysC	-1.076136555	-2.844046667	-2.823729942	-2.585087576	2.89E-03	1.59571E-29	3.51E-08	4.81E-22	adenylyl-sulfate kinase
cysH	-0.810337901	-1.220034657	-1.717382626	-1.623751167	0.001854202	4.26177E-14	1.41E-15	2.50E-18	phosphoadenylyl-sulfate reductase
cysJ	-3.059700493	-6.108961003	-6.835016226	-7.540692525	6.08409738861663E-149	0	0	0	assimilatory sulfite reductase (NADPH) hemoprotein subunit
dppC	7.047504793	6.964496284	8.69982542	7.598959054	2.69E-11	1.56142E-20	4.45E-17	1.59E-24	ABC transporter permease
dppB	6.121912495	6.694401129	5.971162446	7.392521403	9.57E-12	1.35891E-35	7.15E-27	1.36E-42	ABC transporter permease
nikD	1.810284576	2.242316951	2.773873783	2.269883077	3.55E-03	1.41E-04	4.06E-07	1.36E-04	nickel import ATP-binding protein NikD
nikA	1.703536393	2.280646474	0.985127072	1.908966194	1.59E-02	3.24E-04	7.00E-05	4.58E-03	nickel ABC transporter substrate-binding protein
glnH	0.541560604	7.031845988	6.376798605	2.759022641	4.89E-03	3.83704E-16	6.81E-13	1.10E-20	transporter substrate-binding domain-containing protein
glnP	1.155679141	7.923835618	7.20403942	8.400164886	3.57E-03	1.25715E-10	1.39E-08	5.43E-12	amino acid ABC transporter permease
fdhA	3.463657233	4.47500859	3.942188826	3.534145876	3.06E-116	1.3705E-281	2.65E-230	1.41E-166	formaldehyde dehydrogenase, glutathione-independent
recG	-0.774378527	-0.994544489	-0.669876632	-0.438016978	1.09E-05	3.49222E-09	1.30E-04	1.06E-02	ATP-dependent DNA helicase RecG
ruvB	0.478777316	0.548259153	0.783214369	1.006505744	1.26E-02	1.39E-03	5.88E-06	3.45E-09	Holliday junction branch migration DNA helicase RuvB
rapK	2.358342872	2.408519348	2.234313185	2.156843741	3.21E-57	6.69529E-83	1.73E-97	2.94E-79	tetratricopeptide repeat protein
rapA	3.357874757	6.083973534	6.265107655	5.839415956	3.28E-93	0	0	0	tetratricopeptide repeat protein
rapH	1.003512447	2.802063263	3.191821429	3.623873319	3.62E-08	4.64756E-47	3.28E-75	1.49E-102	Rap family tetratricopeptide repeat protein
spo0F	2.373904104	3.202637389	3.091526088	3.438613028	6.92E-23	8.31173E-52	1.37E-52	3.68E-64	response regulator
spo0A	1.690739392	3.137617097	3.012487208	3.624065689	2.21E-27	3.8432E-151	1.94E-144	1.48E-177	sporulation transcription factor Spo0A
